# The Perils of Medical Practice

**Published:** 1897-07-24

**Authors:** 


Vol. XXII.—No. 565.] [July 24, 1897.

### The Perils of Medical Practice.

A large proportion of young medical men, and especially of those who engage early in general practice, have occasion to regret the abolition of the old system of apprenticeship. When this system was in force, as it was, say, fifty years ago, the boy from school did not proceed at once to a hospital, but passed through an intermediate stage of pupilage or apprenticeship. As regards the acquirement of medical knowledge, the system worked more or less well, according to circumstances, inasmuch as some practitioners were better qualified than others to superintend a course of elementary professional study. However this might be, there were certain things which the pupil could scarcely avoid learning; and, among these, were the methods of conducting medical work. He would early be put upon his guard, by precept and by example, against the troubles which may arise out of attendance upon neurotic or hysterical women; and he would both see the nature, and learn something of the value, of the precautions which men of experience use in dealing with them. He would learn how to avoid compromising situations; and how to deal with them if they could not be avoided. Apprenticeship is dead, killed by the demand for a better and more prolonged general education than was formerly thought necessary; but it might be well if the Medical Council and the authorities of the medical schools would consider whether it might not be possible to arrange for systematic teaching of the principles of medical conduct. A boy of seventeen, who goes from a general to a medical school, and from a medical school into practice, may work with great credit at the recognition and treatment of disease, while he remains untaught and unskilled in the management of the patients in whom the diseases are manifested.

The famous question of Henry IV., "Who is she?" will very frequently arise in relation to the scrapes and perplexities of practice. Of course, the directly sexual relation is the origin of the most serious difficulties and entanglements; but a mere relaxation of caution in replying to the dexterous inquiries of a woman about her female neighbours may often be the occasion of a storm which it is far more easy to raise than to allay. The skilful querist who succeeds in obtaining from a young doctor some particulars about the illness of her friend will be very careful not to place him in a position in which he can disclose any confidences with regard to herself; and, as a mere matter of making way in practice, nothing is more important than the recognised habit of refusing to gratify curiosity. It will be remembered that not long ago a medical man was cast in damages for having informed a mistress, quite correctly, that her servant was pregnant, he not having obtained permission from the patient to make disclosures concerning her state; and many somewhat similar cases have been recorded. The recent trial in the Divorce Court, in which a gentleman employed as *locum tenens* was placed in the position of co-respondent with reference to the drunken wife of his employer, is full of interest and instruction, because, in all such conditions, it is the first step which costs, and it is only in the discretion by which the first step may be avoided that safety is to be found. A young doctor, as soon as he realises the existence of sex in a female patient, or as soon as a female patient shows any indication of recognising it in him, will, if he be wise, manage never to have any professional interview with her within closed doors, except in the presence of another woman. Nor will he accompany her on moonlight walks in unfrequented places, where he and his companion are sure to be seen by somebody who will be likely enough to put the worst possible construction upon their proceedings. Such situations, moreover, have often furnished occasions for that form of extortion which the French call *chantage*, and for which we have no equivalent English word or phrase, unless it be the absolutely inappropriate one of "blackmailing." Blackmail was simply an insurance premium paid by Scotch farmers near the Highland line to some neighbouring chieftain, who, in return for the payment, guaranteed the safety of their cattle against thieves. The recipient of blackmail fulfilled his contract, and made good any losses he was unable to prevent. The practitioner of *chantage* keeps no promises, and becomes more greedy the more frequently he is bought off, while the fact of having bought him off, even once, would be accepted by many people as a confession of guilt on the part of his victim. The doctor who once yields to extortion of any kind is lost, and whatever may be the circumstances, or whatever indiscretion may in consequence be disclosed, his only safety is not only to refuse compliance, but at once to hand over the would-be extortioner to the grasp of the law. In taking this course, the services of the Medical Defence Association will often be of the highest value, and timely recourse to their experienced legal advisers would have saved much misery to many weak but otherwise estimable men.

